# Endorsement of metaphysical idealism mediates a link between past use of psychedelics and wellbeing

**DOI:** 10.1038/s41598-024-63687-4

**Published:** 2024-06-10

**Authors:** Jussi Jylkkä, Andreas Krabbe, Patrick Jern

**Affiliations:** Department of Psychology, Åbo Akademi University, Turku, Finland

**Keywords:** Psychedelics, Metaphysical belief, Idealism, Materialism, Worldview, Wellbeing, Psychology, Human behaviour

## Abstract

It has been proposed that psychedelics promote wellbeing through spiritual-type transformations, involving changes in metaphysical beliefs. Past empirical research shows a link between the use of psychedelics and the endorsement of non-physicalist metaphysical beliefs. However, non-physicalist beliefs encompass a wide range of metaphysical ideas, and their links to wellbeing and psychedelics use remain unclear. We utilized a cross-sectional Internet survey to probe the metaphysical beliefs of participants (*N* = 701) with past experience of classical psychedelics, using a novel 42-item questionnaire (Core Metaphysical Beliefs, CMB), encompassing a wide range of metaphysical beliefs. Factor analysis of CMB revealed two factors, Idealism and Materialism. In network analyses, Idealism was linked to psychological insight in a past psychedelic experience (*E* = 0.24) and average use of psychedelics (*E* = 0.16), and predicted wellbeing (*E*s = 0.13 and 0.22). Mediation analyses showed an indirect link from past psychedelics use through Idealism to wellbeing (*p*s ≤ .005). Non-Physicalist Beliefs or Materialism were not significant mediators. The results indicate that Idealism specifically, not non-physicalist beliefs generally, mediate a link between the use of psychedelics and wellbeing. Future research is required to establish whether the link is causal, and to understand what the Idealism factor means.

## Introduction

The use of psychedelics has been linked with the endorsement of non-physicalist metaphysical beliefs^1–3^, which have also been associated with increased wellbeing^4^. Physicalism is the metaphysical view which holds that all of reality ultimately consists of non-conscious physical processes. Non-physicalist views include panpsychism, which considers consciousness as a fundamental aspect of everything that exists, or idealism, which entails that nothing but consciousness exists. Metaphysical beliefs pertain to the fundamental nature of reality and form the core of one’s worldview, that is, the way a person sees the world and one’s place in it^5–7^. It has been proposed that spiritual-type transformations which involve shifts in metaphysical beliefs could be a central underlying mechanism of positive outcomes in psychedelic-assisted therapy^8–10^. However, research on metaphysical belief changes associated with the use of psychedelics is scarce and has focused on non-physicalist beliefs, which encompass a wide range of metaphysical ideas. In this study, our aim was to examine the structure of metaphysical beliefs in a sample of participants with experience of classical psychedelics (e.g., psilocybin, lysergic acid diethylamide (LSD), dimethyltryptamine (DMT), or mescaline). Moreover, we examined the associations between past experience of psychedelics, metaphysical beliefs, and psychological wellbeing and illbeing.

In terms of predictive coding neuroscience^11^, such as the free energy principle^12^, metaphysical beliefs can be considered as *high-level priors*, that is, cognitively fundamental and relatively stable assumptions about the reality which shape our perceptions and interpretations about the world. According to the REBUS model concerning the action of psychedelics on the brain and cognition, psychedelics temporarily diminish the precision-weighting of high-level priors, enabling their revision through increased endogenous or exogenous bottom-up signaling^13^. This could facilitate the questioning and revision of metaphysical beliefs^4^. Hypothetically, such changes could become permanent if they can be integrated with the rational mind^14^. This process could be facilitated by increased neuroplasticity due to the neuropharmacological effects of psychedelics^15,16^.

Experimental research supports the notion that lay people in fact possess implicit metaphysical beliefs about, for example, the possibility of afterlife^17^, separateness between consciousness and the physical world (i.e., metaphysical dualism)^18,19^, the relationship between mind and matter generally (i.e., idealism, dualism, and materialism)^20,21^, and about the existence of free will^22–25^. Such beliefs have been linked to, for example, health behaviors^23^, aggression and prosocial behavior^24^, as well as tendency to cheat^26^. More broadly, metaphysical beliefs can be argued to be central for cognition^5^. For example, metaphysical beliefs about the interconnectedness and relatedness of entities and the insubstantiality of individual things could underlie a *holistic cognitive style* where rational, analytical thinking is devalued. By contrast, the metaphysical belief in distinct entities with essential properties can be linked to an *analytic cognitive style* where logic and reasoning are primarily used to solve problems^5^.

In assessing what types of metaphysical belief changes psychedelics could catalyze, we may consider the phenomenology of psychedelic experiences. Psychedelics are known to facilitate mystical-type experiences^27–31^. The depth of the mystical-type experience, typically assessed in retrospect with questionnaires such as the Mystical Experience Questionnaire (MEQ30)^27^ has been linked to the outcomes in psychedelic-assisted therapy^32,33^. Such experiences include several metaphysical elements (e.g., perceived insights into the fundamental nature of reality), which could lead to sustained changes in metaphysical beliefs.

Mystical-type experiences are often conceptualized based on the work of Walter Stace, who derived the core components of such experiences by analyzing reports of mystics across religions and cultures throughout history^34^. The most central feature of mystical-type experience is a sense of unity where the subject feels that they lose their usual sense of self and become one with something larger. This unity is often experienced as contact with the “ultimate nature” of reality, that is, something that transcends that what can be perceived or conceived intellectually. The transcendent reality can be conceptualized as a type of living presence or consciousness in all that exists, an idea resonating with metaphysical panpsychism (i.e., the belief that consciousness is a fundamental aspect of the reality) or idealism (i.e., the belief that nothing but consciousness exists). Moreover, mystical-type experiences typically involve positive mood and sense of sacredness, the feeling of being in contact with something divine or spiritual. Mystical-type insights are typically experienced as true or noetic^35^, but also as ineffable or impossible to capture in language. From a philosophical perspective, mystical-type experiences have been argued to enable a *sui generis* type of knowledge, different from ordinary, conceptual or representative knowledge^36,37^.

Given the noetic quality of mystical-type experiences, it is plausible that they could lead to sustained changes in metaphysical beliefs. The metaphysical core of a mystical-type experience, as conceptualized by Stace, can be summarized as the thesis that there is a fundamental, unitary nature of reality that transcends the perceivable one, and is in some sense conscious and sacred. It can be hypothesized that such acute metaphysical insights, if they can be integrated with the rational mind and one’s worldview, can maintain the positive effects of mystical-type experiences in the long term^14^, in line with transpersonal approaches to psychological wellbeing^8–10^.

Several studies have found that the use of psychedelics is associated with increases in spirituality, broadly defined^30,31,38–41^. However, the focus in these studies has not been on metaphysical beliefs. Lerner and Lyvers focused more directly on metaphysical beliefs and found that recreational psychedelic drug use was associated with mystical-type metaphysical beliefs, such as belief in universal soul, unity of all things, illusory nature of physical existence, and the existence of a transcendental reality^1^. Timmermann et al. focused specifically on metaphysical beliefs in two longitudinal studies, one conducted online, and the other as part of a clinical placebo-controlled trial^4^. The use of psychedelics facilitated a shift from physicalism to non-physicalist beliefs, such as the belief that there exist other realms or dimensions beyond the physical world (transcendentalism), or that consciousness is a fundamental feature of the universe (panpsychism), or that mind and matter are distinct realms of existence (dualism). Again, belief in physicalism was reduced. The belief changes were maintained up to six months and were positively associated with mental wellbeing.

Nayak and Griffiths asked participants in retrospect about how a single belief-changing psychedelic experience had changed their belief in the existence of consciousness in non-human animals, other living organisms, and inanimate objects^2^. Attributing consciousness to both living and non-living entities was increased, which may reflect higher endorsement of panpsychist ideas. In a subsequent study using the same approach, Nayak et al. assessed changes in a wider range of beliefs with a larger set of questions^3^. The beliefs were categorized in factor analysis into Dualism (e.g., “The body is material and the mind is immaterial” or “The mind and the brain are totally different things”), Paranormal/spirituality (e.g., “It is possible for some people to predict future events” or “Non-physical conscious entities (e.g., souls, angels, spirits) exist”), Panpsychist beliefs (i.e., belief in the presence of consciousness in simpler organisms such as insects or fungi, or even non-living entities such as rocks), and Superstition (e.g., “If you break a mirror, you will have bad luck”). Beliefs in all these categories, except for Superstition, was rated to be increased after the belief-changing psychedelic experience.

In sum, the previous studies have found associations between the use of psychedelics and endorsement of non-physicalist metaphysical beliefs, which have been linked to increased wellbeing. However, the concept of non-physicalism is broad and encompasses a wide range of metaphysical positions. Moreover, some of the mentioned studies have conflated metaphysical beliefs and other types of beliefs, such as paranormal ones. The two need to be kept distinct: metaphysical beliefs are typically abstract and non-falsifiable^42^ and form the core of a person’s worldview, whereas paranormal beliefs in, for example, telekinesis or telepathy, are more concrete and specific and can, at least in principle, be falsified. It could be argued that such paranormal notions contradict scientific knowledge, so that belief in them can be considered as epistemically problematic or even delusional. As argued extensively by philosopher Chris Letheby, psychedelically facilitated false beliefs are problematic even if they would increase psychological wellbeing^43^. By contrast, the truth or falsity of metaphysical beliefs is notoriously difficult to evaluate, as several different metaphysical theories can be compatible with scientific facts^44,45^. Metaphysical beliefs can be considered as, at least to some extent, matters of taste or personal preference. Thus, metaphysical beliefs are arguably epistemically more innocent than other types of beliefs, and could be more plausible as catalysts of therapeutic change in the context of psychedelics. Moreover, focus on metaphysical beliefs is motivated by their hypothesized centrality for worldviews^6^ and cognition generally^5^, which renders them more fundamental than beliefs in, say, paranormal phenomena.

In the present study, our aim was to find out what types of metaphysical beliefs are associated with the use of psychedelics in a sample of participants with at least one psychedelic experience in their past (i.e., an experience facilitated by a classical serotonergic psychedelic). We utilized a novel questionnaire entitled Core Metaphysical Beliefs (CMB) which consisted of 42 items pertaining to a wide range of metaphysical ideas. The goal was to (1) assess the structure of metaphysical beliefs in a sample of participants with experience of psychedelics. Moreover, we aimed to (2) examine whether metaphysical beliefs are associated with past psychedelic use, as well as to (3) examine the associations between endorsement of metaphysical beliefs and mental well-being.

## Method

### Procedure

An online questionnaire was distributed internationally on the crowdsourcing network Prolific.co and targeted people with at least one experience of classical psychedelics. The respondents were required to be at least 18 years old. The recruitment proceeded in two phases: first, a prescreen study to identify people with experience of classical psychedelics; second, the study proper where only eligible participants identified in the prescreen were invited. Completion of the prescreen study took c. 1 min and the compensation was £0.15; completion of the proper study took c. 35 min and the compensation was £4.50. The study was approved by the Ethics Board of the Departments of Psychology and Logopedics at the Åbo Akademi University, Finland (decision number 22/2022). Informed consent was applied from each participant and all methods were performed in accordance with the relevant guidelines and regulations (including the declaration of Helsinki and the General Data Protection Regulation).

The present study was done as a part of a larger preregistered data collection that involved several studies (https://osf.io/pbcvq). In the present article, we combine two studies that were planned to be conducted and published separately (Studies 2 and 3 in the preregistration). When the preregistration was made, the research question of the present study was not clearly formulated, and we deviated from the preregistration in that we included psychological wellbeing and illbeing in the analyses. Moreover, we had planned to conduct both exploratory factor analysis (EFA) and confirmatory factor analysis (CFA) of the CMB, but the CFA was omitted as we considered it as undesirable to conduct both EFA and CFA on the same dataset^46^. Moreover, sample size did not permit splitting the sample into two subsamples.

### Materials

#### Core metaphysical beliefs (CMB)

We used 42 statements, rated on a 1–7 Likert scale (from strongly disagree to strongly agree), presented in Table 1. The items represented 14 metaphysical ideas. We included both mystical-type and other metaphysical ideas to attain a more comprehensive picture of the structure of metaphysical beliefs. We did not expect all the 14 ideas to form psychometrically distinct constructs: although philosophically somewhat distinct, they could be treated by non-philosophers as reflecting similar notions (e.g., panpsychism and idealism may be conflated by laypersons).

**Table 1:** The statements (items) in the Core Metaphysical Beliefs (CMB) and the metaphysical constructs that they were intended to tap onto, as well as item names. Items marked with asterisk (*) were intended as inverted items.

Item	
1. Ultimate nature	
Ultimate1	There is a fundamental nature of reality beyond what can be perceived
Ultimate2	The perceivable world is merely a reflection of a deeper reality
Ultimate3	There is nothing besides what can be observed*
2. Positive ultimate nature	
Pos_Ultimate1	The fundamental nature of reality is something sacred
Pos_Ultimate2	Ultimately, everything is some kind of energy or light
Pos_Ultimate3	Love is the fundamental nature of reality
3. Materialism	
Materialism1	There is nothing but the physical
Materialism2	Consciousness is just neurons firing
Materialism3	Ultimately, everything consists of the entities or processes that physics describes
4. Dualism	
Dualism1	Mental phenomena are not physical
Dualism2	The mind can exist independently of the physical
Dualism3	Mind and matter are distinct aspects of reality
5. Scientism	
Scientism1	Science is the only way to know reality
Scientism2	Nothing exists unless it can be scientifically proven
Scientism3	Science can reveal all there is to know about reality
6. Moral realism	
Moral_realism1	Nothing is absolutely good or bad*
Moral_realism2	Some things are valuable independently of people’s conceptions
Moral_realism3	Some actions are objectively good or bad
7. Monism	
Monism1	All is One
Monism2	Everything is interconnected
Monism3	All that exists are forms of one single kind of stuff
8. Idealism	
Idealism1	The mental is more fundamental than the physical
Idealism2	Nothing exists outside mind
Idealism3	All there exists is forms of consciousness
9. Panpsychism	
Panpsychism1	All entities are to some extent sentient or conscious
Panpsychism2	Consciousness is a fundamental aspect of the universe
Panpsychism3	All matter also has a mental aspect
10. Process/relational ontology	
Process1	Relationships are more fundamental than things
Process2	The reality is fundamentally a constant flux
Process3	Ultimately there are no things but processes
11. Simulationism	
Simulationism1	The world we perceive with the senses is illusory
Simulationism2	The observable reality is like a hallucination
Simulationism3	We create reality in our minds
12. Free will vs determinism	
Free_will1	We can freely decide how to act
Free_will2	All our actions are completely determined by physical causes*
Free_will3	We can actively choose how to act in a given situation
13. Teleology	
Teleology1	Everything happens for a purpose
Teleology2	Life has an ultimate meaning
Teleology3	The Universe unfolds towards a goal
14. Multi / single perspectivism	
Multi_persp1	There is a single true worldview and others are false*
Multi_persp2	There are many valid views of the same world
Multi_persp3	Multiple conflicting worldviews can be true at same time

The statements were chosen to reflect prominent metaphysical theories (e.g., Materialism, Idealism, Panpsychism, Dualism, Monism, or Process metaphysics). Some of the items pertained more broadly to the notion of ultimate nature of reality (i.e., hidden nature of reality beyond the observable) and its positive nature, as this is a central component in mystical-type experiences. We also included a position labeled as “Simulationism”, which was intended to tap on the general Kantian idea that the perceivable reality is somehow illusory. Moreover, we included epistemological notions such as scientism, the idea that science is the only or primary way to know reality^47^, as well as “multiperspectivism”, which entails that several views of the world can be simultaneously true. Free will was included as a specific metaphysical topic, as it has been previously associated with psychedelics use^4^. Finally, we included Teleology, which has been historically a prominent metaphysical theory (cf. Aristotle) and has been linked to the use of psychedelics and mystical-type experiences^3^. Moral Realism was also included. Although ethics is often considered as distinct from metaphysics, metaethical theses such as moral realism pertain not only to what is right or wrong, but also on whether moral facts are objective, mind-independent facts of the reality.

#### Metaphysical beliefs questionnaire (MBQ)

The MBQ was included as a comparison scale for CMB, as it has been previously used to assess metaphysical belief changes related to psychedelics^4^. It consists of nine questions intended to tap on various metaphysical beliefs, assessed on a seven-point Likert scale and yields a single factor, Non-Physicalist Beliefs (NPB). The NPB average score was used in the present study.

#### Holmes et al. worldview scale

The Holmes et al. worldview scale^7^ was used as a comparison scale for CMB and it taps on worldview in a broader sense. It contains 35 items, assessed with 1–5 Likert scale. There are three main factors, Spirituality, Naturalism, and Agnosticism. Each factor contains subfactors, for example, the Spirituality scale includes factors pertaining to spiritual practices and belief in God, as well as other spiritual beliefs. The Naturalism scale consists of both physicalism as a metaphysical thesis as well as scientism as an epistemological thesis. In the present study, we utilized the average scores of the Spirituality and Naturalism scales. The Agnosticism factor was not analyzed in the present study as it mainly does not pertain to metaphysical beliefs.

#### Measures of past psychedelic experience

The mystical experience questionnaire (MEQ30)^27^ was used to assess the degree of mystical-type aspects of the single most meaningful past psychedelic experience (henceforth the Experience). The items in MEQ30 are based on Pahnke’s mysticism scale, which in turn derives from Stace’s definition of the core features of mystical experience. The scale consists of four factors, Mystical, Positive mood, Transcendence of time and space, and Ineffability. Agreement with the items are rated on a six-point Likert scale from zero to five. In the present study, we used the average of all items.

In addition to MEQ30, we used the Psychological Insight Questionnaire (PIQ)^48^ to assess the nature of the Experience. The PIQ was included to assess to what extent the metaphysical beliefs are specifically related to the mystical-type features of the Experience, in contrast to other features that can be personally meaningful or transformative. The PIQ consists of 28 items rated on a Likert scale from zero to five. It consists of two factors, awareness of avoidance and maladaptive patterns (AMP) and insights about goals and adaptive patterns (GAP). In this study, we used the average of all items.

The frequency of past use of psychedelics use was probed by asking the participant how many times they had used psychedelics in their life in total (“Cumulative frequency”, answered by specifying the number of times in an open field); the time since the last experience (“Time since”, given in months); how much they use psychedelics on average (“Average use”, answered on the following scale: “Never” (0), “Once every four years or less often” (1), “Once every three years” (2), “Once every two years” (3), “Once a year” (4), “Few times a year” (5), “Monthly” (6), “Weekly” (7), and “Daily” (8)); and average dose (“Average Dose”, answered on a three-point scale from “small” (1) through “medium” (2) to “strong” (3), or “I don’t know”, with examples of typical doses of commonly used substances).

#### Psychological wellbeing measures

For measuring mental wellbeing, we used The Warwick-Edinburgh Mental Wellbeing Scale (WEMWBS), covering hedonic and eudaimonic wellbeing, functionality, and interpersonal relationships^49^. The scale consists of 14 items, rated on a five-point Likert scale. Items were averaged to produce a single score.

The Peace of Mind Scale (PoMS)^50^ was used as another measure of wellbeing. In contrast to WEMWBS which emphasizes positive emotion, the PoMS focuses on low-arousal positive affect and a sense of peacefulness and harmony. PoMS was originally developed to assess the type of wellbeing valued in the Chinese cultural context but has been applied in the West as well and shown to tap on a unique type of wellbeing^51,52^. PoMS consists of 7 items rated on a five-point Likert scale. The items were averaged to produce a single score.

#### Psychological illbeing measures

Symptoms of depression were assessed with the Patient Health Questionnaire (PHQ-9)^53^ which indexes each of the nine DSM-IV criteria for depression. Participants are asked to rate the severity of their symptoms over the past two weeks on a four-point Likert scale ranging from 1 (“not at all”) to 4 (“nearly every day”). The items were averaged to produce a single score.

Symptoms of anxiety were assessed using the seven-item General Anxiety Disorder Assessment (GAD-7)^54^. Participants are asked to rate the severity of their symptoms over the past two weeks on a four-point Likert scale ranging from 1 (“not at all”) to 4 (“several days”). The items were averaged to produce a single score.

### Statistical analyses

#### Factor analysis

To determine the factor structure of the CMB, we utilized exploratory factor analysis. Following the recommendations of Howard^55^, principal axis factoring (PAF) was used, as it enables detecting latent factors, unlike other EFA methods. Oblimin rotation was used, as we wished to allow the factors to correlate with each other, that is, we could not a priori determine whether the constructs are correlated or not. Problematic items were deleted according to Howard’s 0.4–0.3–0.2 rule, which entails that an acceptable item should load at least 0.40 on the primary factor; load on any secondary factor below 0.30; and that the minimum difference between the primary and secondary factor loadings must be 0.20^55^. Average factor scores were used in subsequent analyses.

#### Convergent validity

To examine the convergent validity of the CMB factors, bivariate correlations were used to compare them to the previously used MBQ questionnaire with the single factor Non-Physicalist Beliefs (NPB), as well as the Spirituality and Naturalism factors from the Holmes et al. worldview questionnaire.

#### Network analysis

Network analysis was used to examine the relationships between past psychedelic experience, the metaphysical belief factors (as assessed with the CMB), and wellbeing. One model was run for variables related to the frequency of past use of psychedelics, and another for the quality of the Experience (i.e., the single most personally meaningful psychedelic experience in the person’s past, assessed in retrospect with the PIQ and MEQ30). In network analysis psychological processes are modelled as a set of interconnected nodes and edges, representing observed variables and statistical relationships between the variables, respectively^56–59^. A Gaussian Graphical Model (GGM) partial correlation network was estimated, with negligible correlations reduced to zero. Technical details of the network analysis are described in the Appendix.

#### Mediation analysis

To examine whether metaphysical beliefs mediate a link between past use of psychedelics and wellbeing, mediation analysis was used. The analyses were conducted in JASP (version 0.18.1), using bias-corrected percentiles and bootstrapping with 1,000 iterations. Standardized estimates were used.

## Results

The data used in the analyses is available at the Open Science Framework (https://osf.io/5vrkn/files/osfstorage). Altogether 2500 participants filled in the prescreen study, of which 1360 had at least one experience of classical psychedelics and were invited to partake in the proper study. Data gathering was stopped at roughly *N* = 700, based on the preregistration and power considerations. As a rule of thumb, factor analysis requires 3–20 observations per variable^60^, and with the 42 CMB-variables, this makes roughly 17 observations per variable, which was deemed sufficient.

Altogether 701 participants completed the survey. Of these, 14 were excluded because their reported lifetime frequency of classical psychedelics use was 0; 29 because their Experience was not facilitated by a classical psychedelic; 16 because they reported having used > 2 psychedelics to facilitate their experience; and 13 because they reported having used other drug(s) than classical psychedelics to facilitate their experience (mainly cannabis). Thus, the final sample was *N* = 629.

The participants were from a wide range of countries, with most respondents from South Africa (*n* = 150), followed by UK (*n* = 111) and Poland (*n* = 74). The Experience mainly took place in a home or home-like environment (70%), followed by nature (9%) and public gathering (e.g., a festival; 9%). Main intention for the Experience was reported as “to relax and enjoy” (60%), followed by “curiosity” (52%). Only 10% and 7% reported a “spiritual” or “therapeutic” intention, respectively. Demographic information as well as information about past psychedelics use and descriptive data of questionnaire responses is presented in Table 2. Gender distribution and drugs used to facilitate the Experience are summarized in Table 3.

**Table 2:** Participant characteristics.

Demographic information	Mean	Std. Deviation	Minimum	Maximum	Cronbach's *α*	
Age	32.25	10.20	19.00	74.00	-	
Education level (1–6)	3.86	0.77	2.00	6.00	-	
Income level (1–5)	2.77	0.89	1.00	5.00	-	
Past use of psychedelics						
Cumulative frequency	7.70	26.72	1.00	500.00	-	
Time since last use (years)	2.93	5.48	0.00	45.83	-	^a^
Average use of psychedelics [0–8]	2.62	1.93	0.00	8.00	-	
Average dose (1–3)	1.42	0.58	1.00	3.00	-	^b^
Mystical Experience Questionnaire [0–5]	2.62	0.94	0.07	4.77	0.95	
Psychological Insight Questionnaire [0–5]	1.82	1.09	0.00	4.57	0.96	
Other questionnaires						
Non-physicalist beliefs (1–7)	4.07	0.96	1.00	6.67	0.73	
Holmes et al. Spirituality factor (1–5)	2.75	0.99	1.00	5.00	0.95	
Holmes et al. Naturalism factor (1–5)	3.21	0.75	1.00	5.00	0.88	
The Warwick-Edinburgh Mental Wellbeing Scale (1–5)	3.31	0.71	1.00	5.00	0.93	
Peace of Mind Scale (1–5)	3.00	0.88	1.00	5.00	0.90	
Anxiety (1–4)	2.05	0.77	1.00	4.00	0.91	
Depression (1–4)	1.90	0.70	1.00	4.00	0.90	

Education level was measured on ordinal scale from 1 to 6 (1 = Primary education; 2 = Lower Secondary education, 3 = Upper Secondary or Vocational education, 4 = University: Bachelor’s degree, 5 = University: Master’s degree, 6 = University: Doctoral degree).

Income level was measured on an ordinal scale from 1 to 5, estimating difference to the average in one's homecountry (1 = Much below average; 2 = Slightly below average; 3 = Average; 4 = Slightly above average; 5 = Much above average).

Average use of psychedelics was estimated on an ordinal scale from 0 to 8 (0 = Never; 1 = Once every four years or less often; 2 = Once every three years; 3 = Once every two years; 4 = Once a year; 5 = Few times a year; 6 = Monthly; 7 = Weekly; and 8 = Daily). Average dose was estimated on an ordinal scale from 1 to 3 (1 = Small; 2 = Medium; 3 = Strong) with a "Don't know" option. Examples of typical doses of commonly used substances were given.

^a^Missingness was 3.

^b^Missingness was 135 due to not knowing the dose.

**Table 3:** Gender distribution and drugs used to facilitate the Experience.

	*n*	*%*
Gender		
Male	362	58
Female	262	42
Other	5	0.8
Experience facilitated by		
LSD	293	47
Psilocybin	339	54
DMT	40	6
5-MeO-DMT	4	0.6
Mescaline	20	3

### Factor analysis of core metaphysical beliefs

To determine the factor structure of the CMB, we utilized exploratory factor analysis. Kaiser–Meyer–Olkin measure indicated adequate sampling (KMO = 0.904) and Bartlett’s test of sphericity was significant (*p* < 0.001), indicating that the data was suitable for factor analysis. The number of factors was chosen based on the scree plot, where a five-factor model was identified as the main “elbow”. The resulting five factors made sense prima facie, so we proceeded to further analysis. Howard’s 0.4-0.3-0.2 rule^55^ resulted in the deletion of 18 items, leaving 24 items. The resulting factors were interpreted as Idealism (*α* = 0.784), Materialism (*α* = 0.873), Free Will (*α* = 0.697), Multiperspectivism (*α* = 0.581), and Monism (*α* = 0.681). Given that the factors Free Will, Multiperspectivism, and Monism had low reliabilities and only consisted of two items per factor, we considered them as redundant and these factor were omitted from further analyses. The items in the Idealism and Materialism factors did not show problematically high (*r*s ≥ 0.8) correlations (all item correlations are presented in Tables A3 and A4 in the Appendix). The final factor loadings are presented in Table 4. The factor scores for Idealism (*M* = 4.29, *SD* = 0.98) and Materialism (*M* = 3.48, *SD* = 1.22) were calculated as the average of the respective items.

**Table 4:** The final factor structure and factor loadings of the Core Metaphysical Beliefs Questionnaire.

Item code		1	2
	1. Idealism (α = 0.784)		
Idealism3	All there exists is forms of consciousness	0.59	0.07
Idealism1	The mental is more fundamental than the physical	0.57	− 0.08
Pos_Ultimate3	Love is the fundamental nature of reality	0.54	− 0.06
Panpsychism3	All matter also has a mental aspect	0.52	− 0.04
Idealism2	Nothing exists outside mind	0.52	0.26
Panpsychism2	Consciousness is a fundamental aspect of the universe	0.51	− 0.16
Dualism2	The mind can exist independently of the physical	0.49	− 0.20
Dualism3	Mind and matter are distinct aspects of reality	0.42	0.01
	2. Materialism (α = 0.873)		
Scientism1	Science is the only way to know reality	− 0.09	0.74
Scientism3	Science can reveal all there is to know about reality	0.05	0.74
Materialism1	There is nothing but the physical	− 0.15	0.73
Scientism2	Nothing exists unless it can be scientifically proven	0.09	0.68
Materialism2	Consciousness is just neurons firing	− 0.11	0.66
Free_will2	All our actions are completely determined by physical causes	− 0.06	0.65
Ultimate3	There is nothing besides what can be observed	0.23	0.63
Materialism3	Ultimately, everything consists of the entities or processes that physics describes	− 0.12	0.61

### Convergent validity

To examine the convergent validity of the CMB factors, they were compared to the previously used MBQ questionnaire^4^ which yields the single factor Non-Physicalist Beliefs (NPB), as well as the Spirituality and Naturalism factors from the worldview questionnaire by Holmes et al.^7^.

The NPB was positively correlated with CMB-Idealism (*r* = 0.62, *p* < 0.001) and negatively but weaker with CMB-Materialism (*r* = − 0.29, *p* < 0.001). The Spirituality factor from the Holmes et al. worldview questionnaire was positively correlated with CMB-Idealism (*r* = 0.59, *p* < 0.001) and negatively with CMB-Materialism (*r* = − 0.38, *p* < 0.001). The Holmes et al. Naturalism factor was negatively correlated with CMB-Idealism (*r* = − 0.29, *p* < 0.001) and positively with CMB-Materialism (*r* = 0.75, *p* < 0.001).

### Network analysis

Two network models were conducted to examine whether past psychedelic use was associated with endorsement of metaphysical beliefs and wellbeing. One model (“Acute model”) included measures of the acute features of the Experience (PIQ and MEQ30) and the other (“Frequency model”) included variables related to frequency of past use (Cumulative frequency, Time since last use, and Average use). In the Frequency model, Average dose was omitted due to large number of participants (*n* = 135) not knowing the average dose. Before model estimation all variables were checked for univariate and multivariate normality using the MVN package^61^. The data deviated from multivariate normality based on Mardia’s skewness for the acute model (*S* = 448.342, *p* < 0.001) and kurtosis (*K* = 6.476, *p* < 0.001) as well as for the frequency model (*S* = 20,029.873, *p* < 0.001) and kurtosis (*K* = 214.844, *p* < 0.001). Therefore, before estimation nonparanormal transformations were performed on the data. To check the robustness of the estimated parameters, bootstrapped 95% CIs were estimated for all the edge weights in both models. The results pointed to relatively narrow CIs indicating robust results (see Figs. A1 and A2 in the Appendix). A stability coefficient of 0.75 was estimated for both models, which exceeded the recommended threshold of 0.5 for psychological networks and points to stable strength centrality estimates^62^. All network estimates as well as raw strength centrality estimates of nodes are presented in the Appendix (see Tables A1 and A2, and Figs. A3 and A4, respectively).

**Figure 1:**
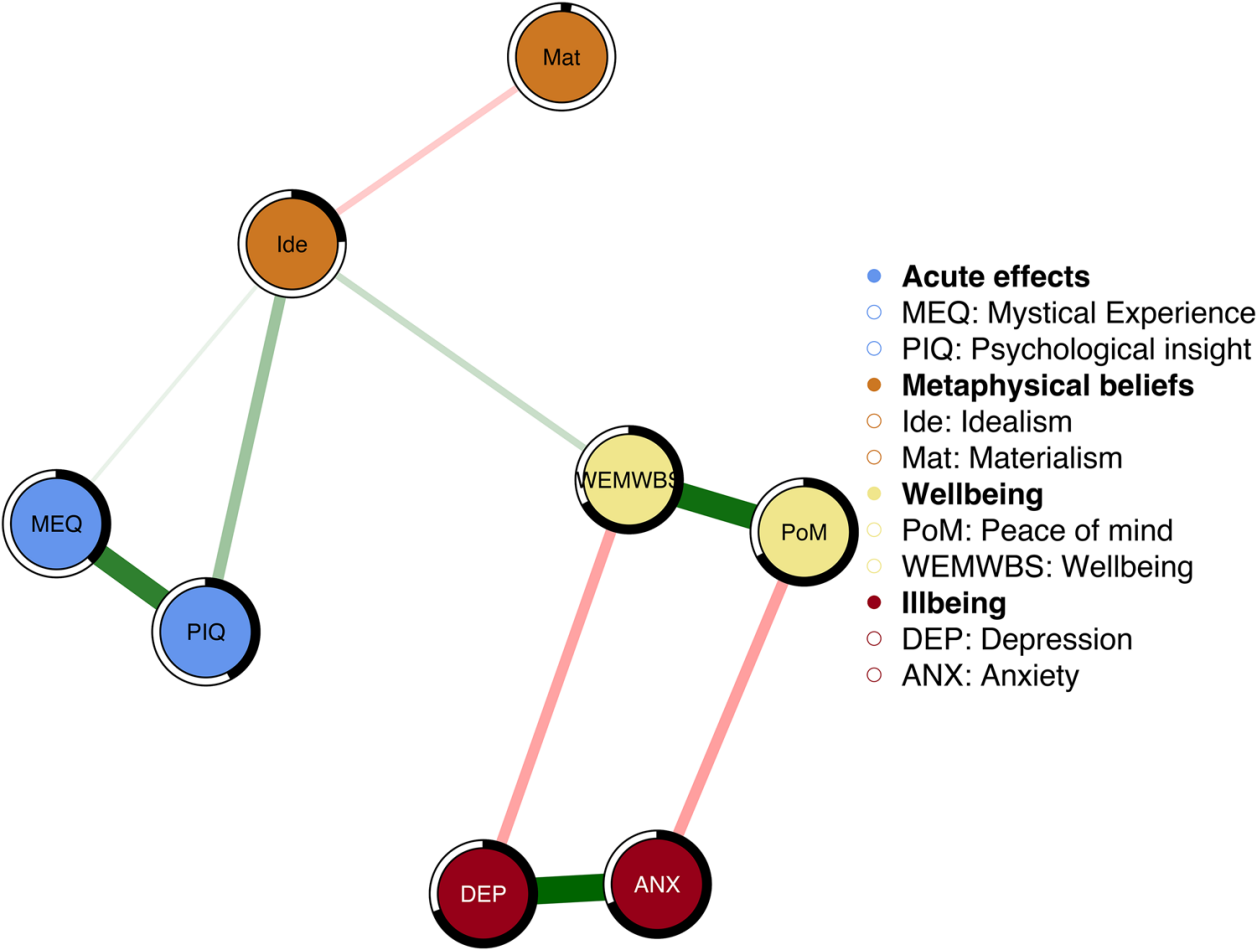
Associations between the features of a past, personally meaningful psychedelic experience (“Acute Effects”), wellbeing/illbeing, and metaphysical beliefs (Materialism and Idealism). Edge thickness depicts strength of correlation. Small correlations were restrained to zero and partial correlations were used. The node wheels indicate how much of an item’s variance is explained by the model.

**Figure 2:**
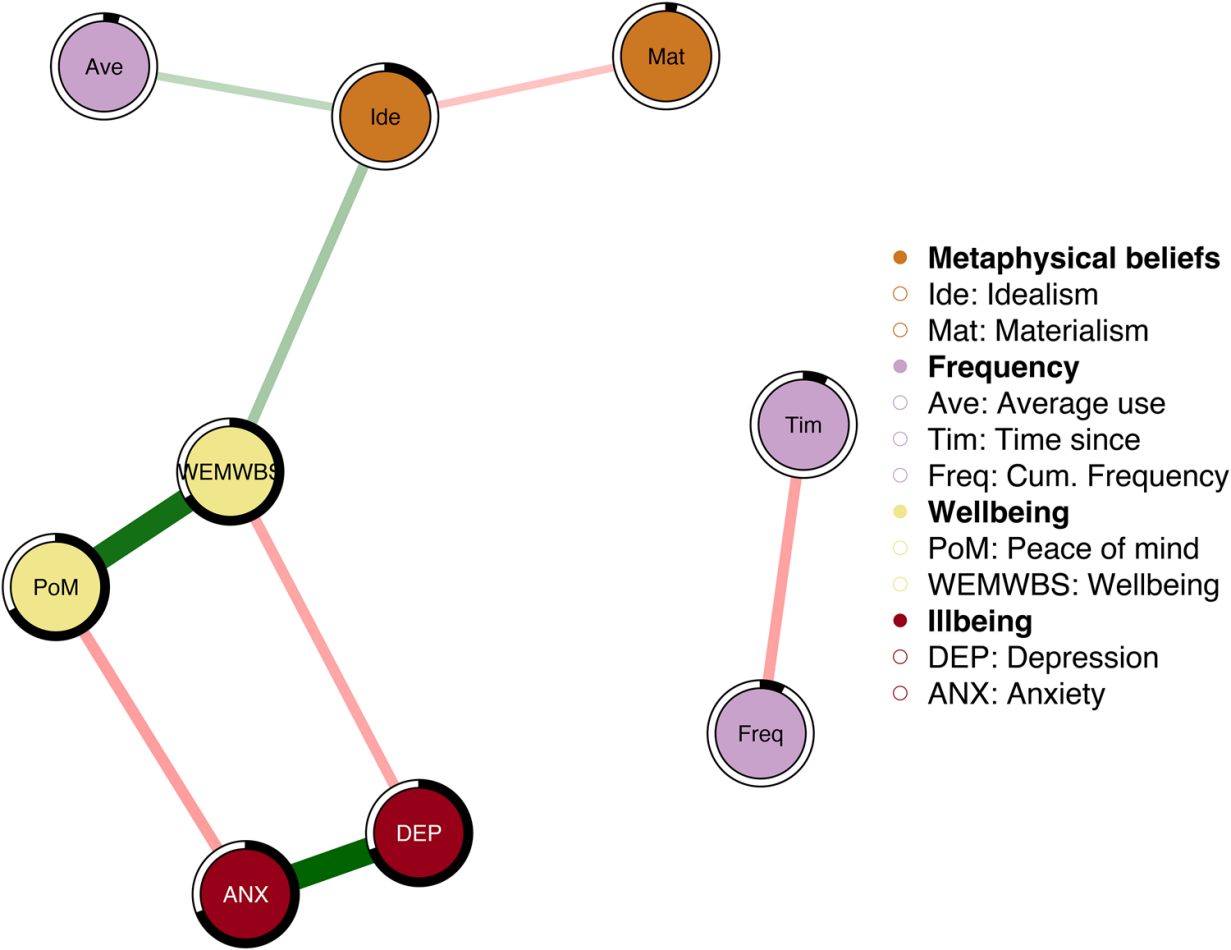
Associations between the frequency of psychedelics use, wellbeing/illbeing, and metaphysical beliefs (Materialism and Idealism). Edge thickness depicts strength of correlation. Small correlations are restrained to zero and partial correlations are used. The node wheels indicate how much of an item’s variance is explained by the model.

**Figure 3:**
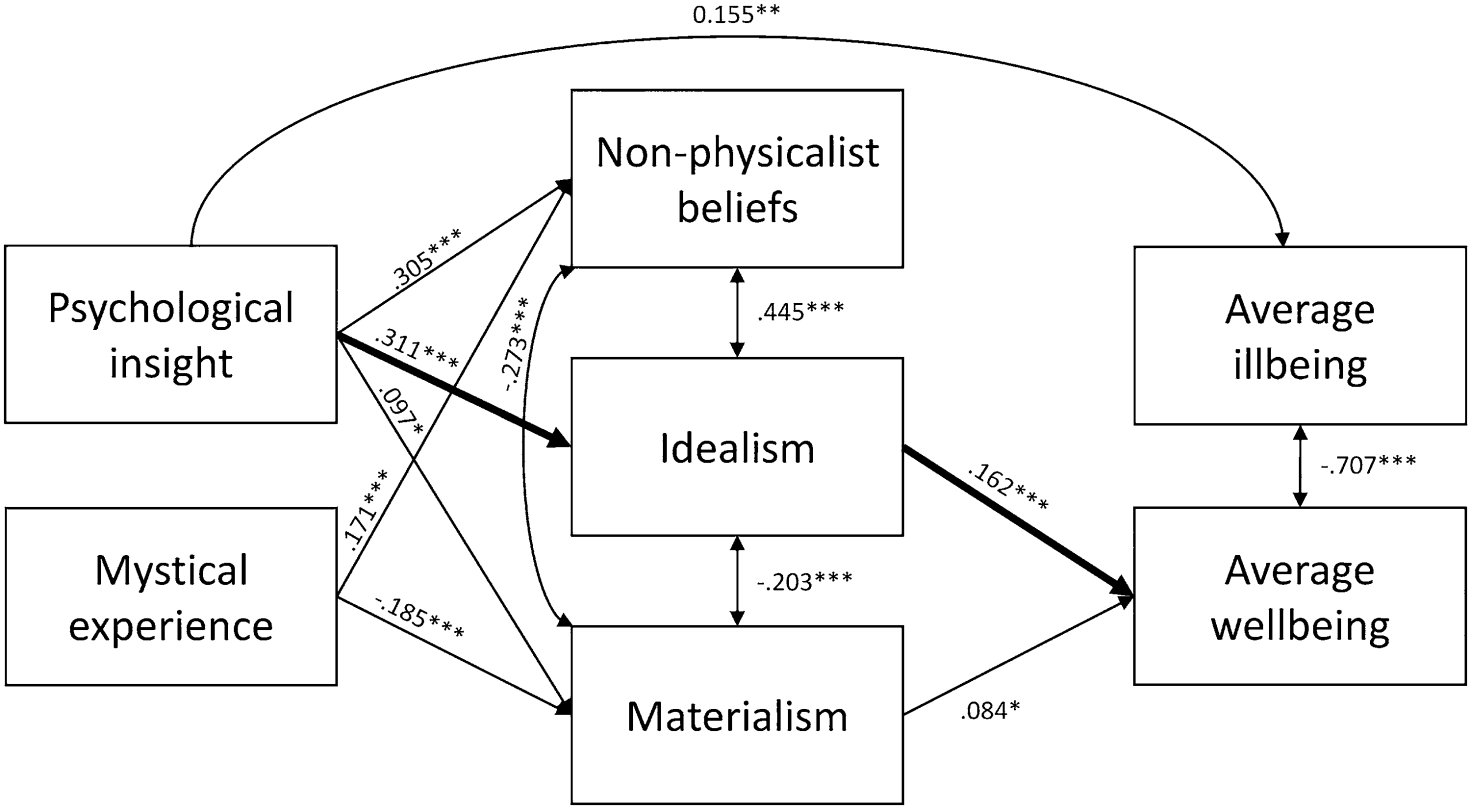
Mediation model with acute features of the Experience as predictors. Only significant path coefficients are displayed. A significant indirect effect from Psychological Insight through Idealism to Average wellbeing was observed, indicated with bolded arrows.

**Figure 4:**
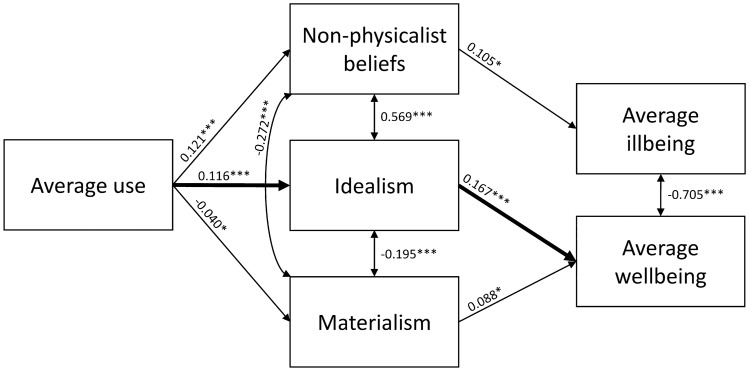
Mediation model with average use of psychedelics as the predictor. Only significant path coefficients are displayed. A significant indirect effect from Average use through Idealism to Average wellbeing was observed (bolded arrows).

In the Acute model (Fig. 1), the variables were clustered as expected, with the acute measures, the wellbeing/illbeing measures, and the metaphysical beliefs forming separate clusters. Idealism and Materialism were negatively associated (*E* = − 0.13). Idealism was associated with psychological insight during The Experience (PIQ) (*E* = 0.24), but the association with mystical-type features (MEQ30) was weak (*E* = 0.06). Further, Idealism was positively associated with psychological wellbeing (WEMWBS) (*E* = 0.13).

In the Frequency model (Fig. 2), the past use variables Time since last use and Cumulative frequency were isolated from the rest of the network, but showed a negative association with each other (*E* = − 0.23). The other variables were clustered as expected. Average use was associated with Idealism (*E* = 0.16), which in turn was associated with psychological wellbeing (*E* = 0.22). Materialism was inversely associated with Idealism (− 0.15).

### Mediation analysis

A post hoc mediation analysis was conducted to examine whether there is an indirect link from past use of psychedelics to wellbeing/illbeing through metaphysical beliefs. This was motivated both theoretically (see Introduction) as well as by the network analysis, which could be taken to suggest an indirect link from past psychedelics use to psychological wellbeing (WEMWBS) through Idealism. Moreover, we wished to examine whether it is Idealism specifically or Non-naturalistic beliefs generally that could mediate the link between psychedelics use and wellbeing; thus, the NPB was also included as a mediator.

The first mediation analysis included the measures about the quality of the Experience (i.e., PIQ and MEQ30). As mediators we included Idealism and Materialism from the CMB, as well as Non-Physicalist Beliefs (NPB), and as the outcome variables Average wellbeing (average of WEMWBS and PoMS) and Average illbeing (average of the depression scale PHQ-9 and anxiety scale GAD-7). No direct effects were observed (*p*s > 0.17), except for an association between PIQ and Average illbeing (*E* = 0.155, *SE* = 0.049, *p* = 0.002), that is, higher occurrence of psychological insight during the Experience predicted more anxiety and depression symptoms. Moreover, there was an indirect effect from PIQ through Idealism to Average wellbeing (*E* = 0.050, *SE* = 0.017, *p* = 0.004). The other indirect effects were non-significant (*p*s > 0.079). Significant effects are summarized in Fig. 3.

The second model included as predictor the frequency variable Average use and as mediators Idealism and NPB, and as the outcome variables the aggregate measures Average wellbeing and Average illbeing. No direct effects of Average use on Average wellbeing or Average illbeing were observed (*p* = 0.48 and *p* = 0.71, respectively). However, there was an indirect effect on Average wellbeing through Idealism (*E* = 0.019, *SE* = 0.007, *p* = 0.004). The other indirect effects were non-significant (*p*s > 0.055). Significant effects are illustrated in Fig. 4.

Given the unexpected positive association between PIQ and Average illbeing, we conducted a follow-up analysis with the subfactors of PIQ, that is, awareness of Goals and Adaptive Patterns (GAP) and Awareness of Maladaptive Patterns (AMP), which were included as predictors. In this analysis, MEQ30 was omitted as predictor to simplify the model and to maintain statistical power on the same level as in previous analyses. As mediators we included Idealism, Materialism and NPB, and as the outcome variable the wellbeing/illbeing aggregate variables. GAP predicted Average wellbeing positively (*E* = 0.37, *SE* = 0.062, *p* < 0.001) and Average illbeing negatively (*E* = − 0.22, *SE* = 0.063, *p* < 0.001), whereas AMP predicted Average wellbeing negatively (*E* = − 0.33, *SE* = 0.064, *p* < 0.001) and Average illbeing positively (*E* = 0.34, *SE* = 0.066, *p* < 0.001). The only significant indirect effect was from GAP through Idealism to Average wellbeing (*E* = 0.035, *SE* = 0.014, *p* = 0.016).

## Discussion

Previous research indicates that the use of psychedelics is associated with the endorsement of non-physicalistic metaphysical beliefs^1–3^, with evidence of a causal link and possible effect on wellbeing^4^. However, non-physicalist metaphysical beliefs encompass a wide range of metaphysical theses. In the present study, our first aim was to explore the structure of metaphysical beliefs in participants with experience of psychedelics, using a more comprehensive questionnaire than previously. Our second aim was to assess whether past experience of psychedelics was associated with the endorsement of metaphysical beliefs, and whether metaphysical beliefs are associated with subjective wellbeing.

### Structure of metaphysical beliefs

Despite the wide range of philosophical ideas that the items in the CMB might reflect, only two reliable factors emerged in the factor analysis, Idealism and Materialism. The Idealism factor consisted mainly of idealism and panpsychism-related items, as well as a notion about the positive nature of the ultimate nature of reality (“Love is the ultimate nature of the reality”). Moreover, the factor included two dualism-related items which could *prima facie* be considered as inconsistent with idealism. However, the dualistic items in the Idealism factor (“The mind can exist independently of the physical” and “Mind and matter are distinct aspects of reality”) can be interpreted in an idealistic framework: if the physical is an illusion, then it follows that mind can exist independently of the physical. Moreover, an idealist can hold that mind and matter are distinct aspects of the reality, the former being part of its fundamental nature, the latter an illusory or superficial aspect^63,64^. However, it could also be that non-philosophers do not possess clearly defined concepts of dualism and idealism, at least as they are commonly philosophically conceived. The Idealism factor could reflect vague and implicit spiritual-type metaphysical beliefs, which could be different from Idealism as a philosophical construct.

The second factor, Materialism, included ontological theses such as “There is nothing but the physical”, but also scientistic items such as “Science is the only way to know reality”. This is in line with the findings of Holmes et al., where metaphysical physicalism and epistemological scientism were both part of a single factor, Naturalism^7^. Moreover, the Materialism factor included “There is nothing besides what can be observed” and “All our actions are completely determined by physical causes”, which were intended as inverted items for other ideas besides materialism, but in their non-inverted form are consistent with materialism.

While the present study was conducted, a similar factor analysis of metaphysical beliefs in laypersons by Rennie et al. was published as a preprint^65^. Their Metaphysics Matrix Questionnaire (MMQ) contained 111 items and exploratory factor analysis yielded eight factors: (1) Religiosity/Spirituality, (2) Physicalism, (3) Transcendency, (4) Panpsychism/Cosmopsychism, (5) Determinism/Fatalism/Low-autonomy, (6) Pseudo-Emergentism, (7) High-autonomy/Moral, and (8) Randomness. Thus, the main difference between the two studies is that the MMQ involves more dimensions of metaphysical beliefs than the CMB. This difference is arguably mainly due to the different kinds of items used, as well as their number: the more different types of items, the more factors can be extracted. Moreover, MMQ did not include many items that tapped specifically on idealism, unlike CMB. Moreover, Rennie et al. did not target participants who had experience of psychedelics, but rather aimed their survey on the general population. It is worth noting that the two main factors (i.e., factors with most items) in both the present study and Rennie et al. correspond to physicalism or materialism and, roughly, spiritual-type metaphysical beliefs.

### Convergent validity

The convergent validity analysis indicated that the CMB factor Idealism measured partly the same construct as the more general Non-physicalist beliefs (NPB) factor of Timmerman et al.^4^, and Holmes et al.’s^7^ broader Spirituality factor. In turn, Materialism measured partly the same construct as Holmes et al.’s broader Naturalism factor. In sum, the Idealism and Materialism factors showed expected correlations which were of medium size, indicating that the factors measure partly the same constructs as the previous questionnaires, although there was substantial unique variance. That is, Idealism cannot be reduced to Non-physicalist beliefs or Spirituality in general.

### The network analysis

The network analysis (Frequency model) on the associations between past experience of psychedelics, metaphysical beliefs (Idealism and Materialism), and wellbeing/illbeing showed a link between Average use of psychedelics and Idealism, which in turn was associated with psychological wellbeing (WEMWBS). The other two frequency-related variables (i.e., Time since last use and Cumulative frequency) were isolated. This could be due to the latter questions being open-ended: the participants manually wrote the number of months since their last psychedelic experience, and manually indicated how many times they had used psychedelics during their lifetime. These numbers may be difficult to estimate and could increase error variance. Regarding the link between Average use and Idealism, a possible interpretation is that psychedelic experiences involve belief-changing aspects, such as metaphysical insights, which lead to sustained increases in Idealistic beliefs. However, it could also be that persons who are inclined towards Idealism are more prone to using psychedelics due to contextual factors. The cross-sectional nature of the data may not afford inferences about causality (although this is not always the case^66^), which calls for longitudinal designs.

In the Acute model, psychological insight during the Experience was linked with the endorsement of Idealism, which in turn was linked with psychological wellbeing, in line with the Frequency model. It was unexpected that it was psychological insight (PIQ) rather than mystical-type features of the experience (MEQ30) that was most strongly associated with Idealism. One might hypothesize that it would be the mystical-type features of the Experience that lead to sustained changes in metaphysical beliefs, not psychological insights. One possible explanation for this finding is that most items in the MEQ30 focus on the phenomenology of the experience, such as experience of “pure being”, oneness, positive mood, and sense of sacredness, not insights per se. The PIQ, by contrast, is directly concerned with insights. Although the PIQ is limited to psychological insights, it is possible that *insightfulness* is a general attribute of psychedelic experiences, such that different types of insights tend to occur simultaneously. It is noteworthy that the PIQ and MEQ30 were strongly intercorrelated and thus measure partly the same construct, which arguably involves both mystical-type features and insight experiences.

In both the network analyses, outside the wellbeing/illbeing cluster it was Idealism that was most strongly associated with psychological wellbeing (WEMWBS). That is, past experience of psychedelics was not directly linked to the wellbeing/illbeing measures. This is consistent with the idea that a mystical-type insight during a psychedelic experience, or insight experience more generally, leads to the endorsement of mystical-type metaphysical beliefs, which in turn maintain psychological wellbeing. However, given the cross-sectional nature of the data, causal conclusions are not directly warranted. It is also possible that people with Idealistic beliefs are more prone to using psychedelics in the first place, or that subjective wellbeing makes a person more likely to believe in Idealism.

### Mediation analyses

The post hoc mediation analysis showed that the link between past psychedelic use and Average wellbeing (average of WEMWBS and PoMS) was mediated specifically by Idealism, not by Materialism, and not by non-physicalist beliefs generally. This pattern of results could be taken to reflect a causal pattern where idealistic beliefs mediate the long-term effects of psychedelic experiences on wellbeing. However, the direction of causality cannot be established in the present cross-sectional research.

Surprisingly, PIQ predicted Average ill-being (average of PHQ-9 and GAD-7), in contrast to previous research^48^. A follow-up analysis revealed that awareness of goals and adaptive patterns (GAP) was associated with wellbeing, whereas awareness of maladaptive patterns (AMP) was associated with illbeing. In this analysis, the only significant indirect effect was from GAP through Idealism to Average wellbeing. This could be taken to suggest that insights about adaptive patterns, such as realizing the value and purpose of one’s life, are linked to endorsing a worldview where the fundamental nature of reality is seen as something valuable and spiritual.

### Implications

The present results are consistent with the hypothesis that altered metaphysical beliefs underlie the long-term effects of psychedelics on wellbeing. This is in line with theorizing by some authors who emphasize the role of spiritual-type transformations in psychedelic-assisted therapy^8–10^. However, such belief changes have also been considered as problematic, as they could contradict the scientific worldview^13,43^. While the question of what kinds of belief changes psychedelics can facilitate is an empirical one, the question of whether these beliefs are rational or delusional is mainly philosophical^45^. It can be argued that supernatural or paranormal beliefs are indeed epistemically problematic, as they can, at least in principle, be empirically falsified and can contradict mature scientific theories (consider, e.g., telepathy or telekinesis). However, metaphysical beliefs are abstract and not empirically falsifiable or verifiable^42^. This includes naturalism or materialism, which are metaphysical, not scientific theories. Thus, one cannot argue that, say, idealistic beliefs are false simply because they contradict naturalism. In sum, metaphysical beliefs can be considered as more epistemically innocent than other types of beliefs associated with psychedelics use, such as paranormal beliefs.

Rather than considering spiritual-type metaphysical beliefs as delusional and something to be avoided, they can be considered as part of the transformative process facilitated by psychedelic experiences. It is intuitively plausible that an insight that the fundamental nature of reality is a type of loving consciousness can be therapeutic for a depressed person who has previously believed that nothing ultimately matters or has any value. If that sudden insight could be transformed into a sustained belief, it could maintain the therapeutic effect. However, a lay person with no knowledge of philosophy could have difficulty in integrating their psychedelic insights with their rational mind and previous knowledge about the reality, including scientific knowledge. In this integration process, philosophy could be beneficial, possibly implemented as a type of philosophical counseling as part of the integration phase of psychedelic-assisted therapy^14^. An important aspect of this process would be to keep epistemically problematic beliefs (e.g., paranormal ones) distinct from genuine metaphysical beliefs, which can be considered as epistemically less problematic (depending on how they are conceptually elaborated).

It is worth noting that while supernatural or paranormal beliefs can be epistemically problematic, this does not entail that they would be problematic for psychological wellbeing. For example, there is evidence that people who have had experiences of communicating with the dead have reduced fear of death and increased spirituality^67^ and people with such experiences find them comforting^68^. More generally, religiosity and spirituality, often involving supernatural beliefs that may contradict scientific knowledge, have been linked with increased wellbeing^69^. Although such beliefs could be argued to be epistemically problematic from a naturalistic perspective, they can be psychologically beneficial. Moreover, it can also be debated whether any conflict exists between supernatural beliefs and scientific knowledge^70^. It is an open question how this tension between possible epistemic problems and psychological benefits should be reconciled. Nevertheless, we consider that it is useful to separate between metaphysical beliefs from other types of beliefs.

### Limitations and strengths

The main limitation of the present study is its cross-sectional design, which makes it difficult to draw conclusions about the direction of causality (but see reference^66^). Moreover, retrospective reports of the Experience could be unreliable due to limited memory, as well as possible biases in recall (e.g., positive present mood could lead to more positive evaluation of the Experience, due to availability bias). This calls for longitudinal studies to address the belief changes related to psychedelics use. Another limitation is that there can exist data quality problems in utilizing online crowdsourcing platforms due to, for example, hasty or unreliable responses^71,72^. This calls for including data quality checks, such as attention tests in crowdsourcing surveys. Unfortunately, the present study did not include such tests, and participants were only screened based on whether their responses regarding past psychedelics use were consistent (as described in Section "Results"). This omission could limit the data quality in the present study. However, unreliable or hasty responding would probably lead to random error, not systematic error, and would mainly weaken the observed effects without biasing them in any specific direction. Future survey studies should control data quality more systematically. A final limitation is that the measure of the core metaphysical beliefs questionnaire (CMB) could be criticized due to omitting specific metaphysical positions, such as the illusory nature of time and space (important in some spiritual traditions), or the illusoriness of the self (central in several Eastern philosophies). On the other hand, the strengths of the present study include a sufficient sample size and participants from various lands and cultures which should result in good generalizability. Moreover, the participants were relatively unexperienced psychedelics users, in contrast to “psychonauts” who may be deeply positioned in psychedelic subcultures, and could be relatively representative of the population of psychedelic users. However, although our study included participants from various countries, we did not control for background factors such as race and ethnicity, which limits inferences about generalizability. Future studies could investigate possible cross-cultural differences in the metaphysical beliefs more closely.

## Summary and conclusions

It has been argued that spiritual-type transformations are an underlying mechanism in psychedelic-assisted therapy, where long-term effects on wellbeing could be driven by sustained changes in one’s worldview and metaphysical beliefs. However, previous research has mainly focused on the broad concept of non-physicalist beliefs. We found that metaphysical beliefs among users of psychedelic drugs can be categorized into two dimensions, Idealism and Materialism, where Idealism can be taken to reflect spiritual-type metaphysical beliefs. Idealism mediated a link between the use of psychedelics and subjective wellbeing. This is in line with the reasoning, common in the literature but rarely empirically tested, that the long-term effects of psychedelic experiences could be mediated and sustained by spiritual transformations, which include changes in metaphysical beliefs. However, assessing the direction of causality would require longitudinal designs or randomized controlled trials.

### Supplementary Information

Supplementary Information.

## Data Availability

All data used in the analyses is available at the Open Science Framework (https://osf.io/5vrkn/files/osfstorage).
