# Supplementary material for: Endorsement of metaphysical idealism mediates a link between past use of psychedelics and wellbeing
Source: Sci Rep. 2024 Jun 10;14:13276. doi: 10.1038/s41598-024-63687-4 (PMC11164882; doi:10.1038/s41598-024-63687-4)
Supplement: Supplementary file 1 — Supplementary Information. [file 41598_2024_63687_MOESM1_ESM.docx]

**Appendix**

**Main article: Endorsement of metaphysical idealism mediates a link between past use of psychedelics and wellbeing**

Jussi Jylkkä, Andreas Krabbe, & Patrick Jern

**1. Network estimation**

The network analysis included three steps: network estimation, network inference (including network properties and centrality), and network stability check. All variables were checked for univariate and multivariate normality using the MVN package (Korkmaz et al., 2014) and only participants with complete data were analyzed. A Gaussian Graphical Model (GGM) partial correlation network was estimated, using the mgm package (Haslbeck & Waldorp, 2015). We used graphical LASSO (Friedman, Hastie, & Tibshirani, 2008) in combination with EBIC model selection (Foygel & Drton, 2010) and a tuning parameter (gamma) set to 0.5 to regularize and optimize the specificity and sensitivity of the network. To obtain the predictability or *R^2^* estimates of each node in the network, which were then included in the graphs, the mgm package (Haslbeck & Waldorp, 2015) was used. Before obtaining predictability estimates, nonparanormal transformations (Liu, Lafferty, & Wasserman, 2009) were performed on the variables to have a marginal normal distribution (Epskamp et al., 2018), using the huge.npn function from the huge package (Zhao, Liu, Roeder, Lafferty, & Wasserman, 2012). To graphically represent the results, we used the modified Fruchterman-Reingold algorithm (Fruchterman & Reingold, 1991) for weighted networks implemented in the qgraph package (Epskamp, Cramer, Waldorp, Schmittmann, & Borsboom, 2012). The bootnet package (Epskamp et al., 2018) was used to estimate the stability and robustness of the estimated network parameters. To check the stability of edges, non-parametric bootstrap 95% confidence intervals (CIs) on the edge-weights with 1000 bootstrap samples were estimated, while the stability of node centrality indices was estimated using the case-dropping bootstrap procedure with 2500 bootstrapped samples.

*Figure A1. Bootstrapped estimates of the network model with the acute features of the Experience*

*
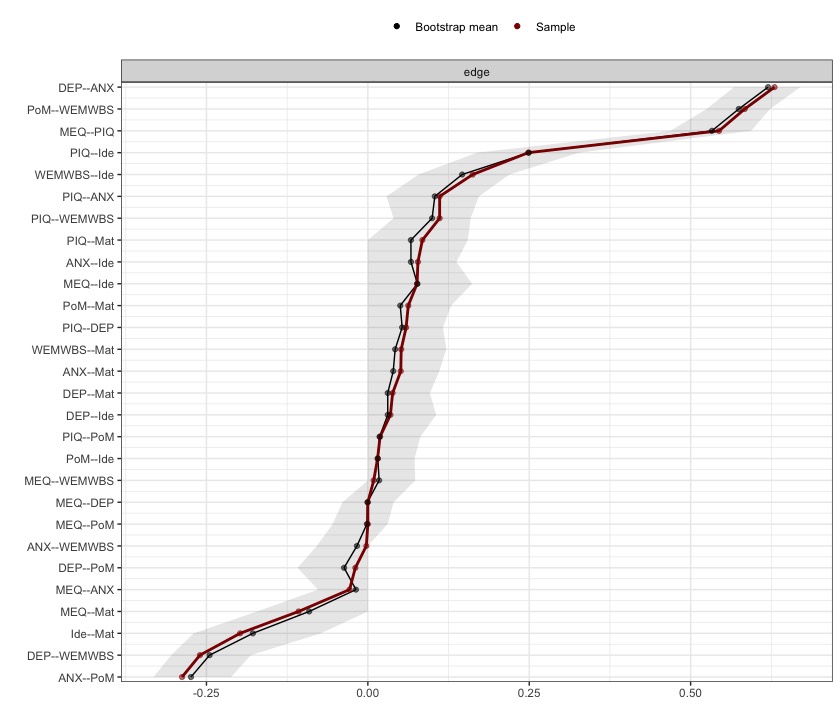
*

*Figure A2. Bootstrapped estimates of the network model with the frequency variables of past psychedelics use.*

*
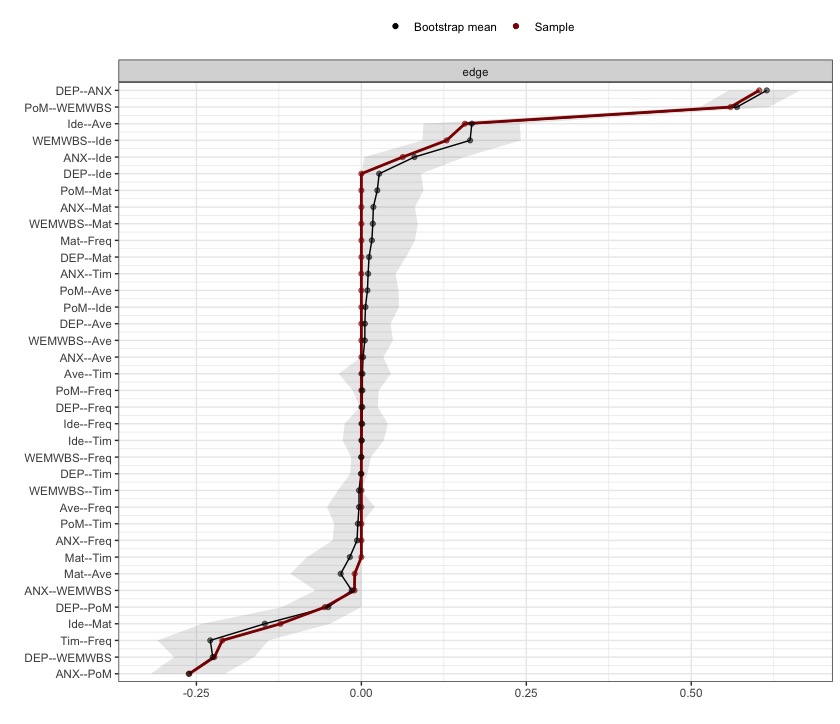
*

*Figure A3. Strength centrality of nodes in the network model with the acute effects*

*
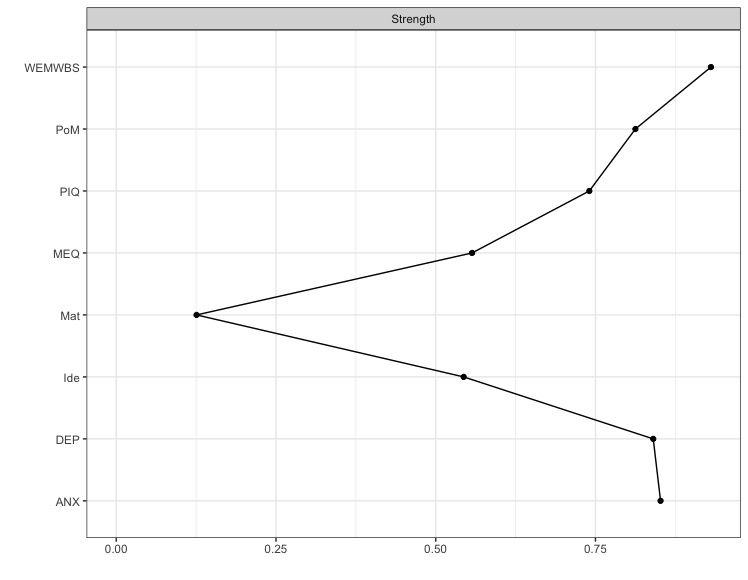
*

*Figure A4. Strength centrality of nodes in the network model with the frequency variables regarding past psychedelics use*

*
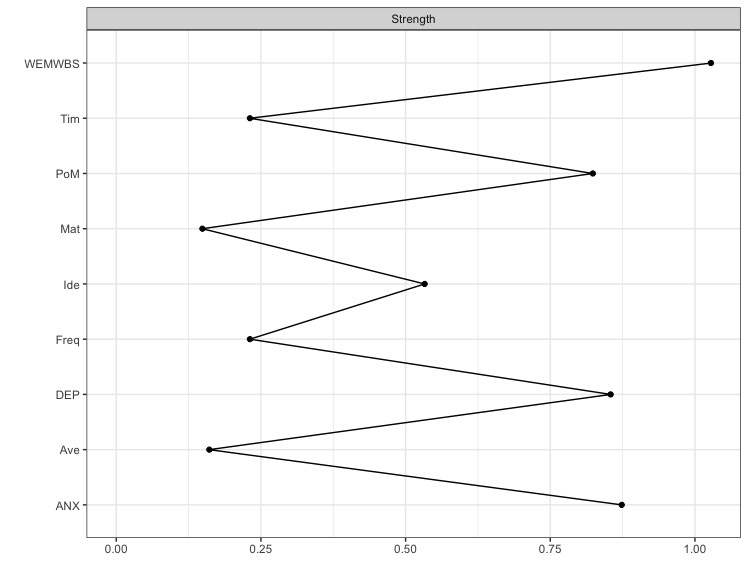
*

*Table A1. Estimates for the network model with the acute features of the Experience*

|  | MEQ | PIQ | DEP | ANX | PoM | WEMWBS | Ide | Mat |
| --- | --- | --- | --- | --- | --- | --- | --- | --- |
| MEQ | 0.00 | 0.51 | 0.00 | 0.00 | 0.00 | 0.00 | 0.06 | 0.00 |
| PIQ | 0.51 | 0.00 | 0.00 | 0.00 | 0.00 | 0.00 | 0.24 | 0.00 |
| DEP | 0.00 | 0.00 | 0.00 | 0.62 | 0.00 | -0.22 | 0.00 | 0.00 |
| ANX | 0.00 | 0.00 | 0.62 | 0.00 | -0.24 | 0.00 | 0.00 | 0.00 |
| PoM | 0.00 | 0.00 | 0.00 | -0.23 | 0.00 | 0.58 | 0.00 | 0.00 |
| WEMWBS | 0.00 | 0.00 | -0.22 | 0.00 | 0.58 | 0.00 | 0.13 | 0.00 |
| Idealism | 0.05 | 0.24 | 0.00 | 0.00 | 0.00 | 0.13 | 0.00 | -0.13 |
| Materialism | 0.00 | 0.00 | 0.00 | 0.00 | 0.00 | 0.00 | -0.13 | 0.00 |

*Table A2. Estimates for the network model with the frequency variables of past psychedelics use*

|  | DEP | ANX | PoM | WEMWBS | Ide | Mat | Ave | Tim | Freq |
| --- | --- | --- | --- | --- | --- | --- | --- | --- | --- |
| DEP | 0.00 | 0.63 | 0.00 | -0.22 | 0.00 | 0.00 | 0.00 | 0.00 | 0.00 |
| ANX | 0.63 | 0.00 | -0.24 | 0.00 | 0.00 | 0.00 | 0.00 | 0.00 | 0.00 |
| PoM | 0.00 | -0.24 | 0.00 | 0.58 | 0.00 | 0.00 | 0.00 | 0.00 | 0.00 |
| WEMWBS | -0.22 | 0.00 | 0.58 | 0.00 | 0.22 | 0.00 | 0.00 | 0.00 | 0.00 |
| Idealism | 0.00 | 0.00 | 0.00 | 0.22 | 0.00 | -0.15 | 0.16 | 0.00 | 0.00 |
| Materialism | 0.00 | 0.00 | 0.00 | 0.00 | -0.15 | 0.00 | 0.00 | 0.00 | 0.00 |
| Average use | 0.00 | 0.00 | 0.00 | 0.00 | 0.16 | 0.00 | 0.00 | 0.00 | 0.00 |
| Time since | 0.00 | 0.00 | 0.00 | 0.00 | 0.00 | 0.00 | 0.00 | 0.00 | -0.23 |
| Cumulative frequency | 0.00 | 0.00 | 0.00 | 0.00 | 0.00 | 0.00 | 0.00 | -0.23 | 0.00 |

**2. Item correlations of the Core Metaphysical Beliefs questionnaire**

*Table A3. Bivariate correlations of the Idealism items*

| **Variable** |  | **Idealism3** | **Idealism1** | **Pos_Ultimate3** | **Panpsychism3** | **Idealism2** | **Panpsychism2** | **Dualism2** |
| --- | --- | --- | --- | --- | --- | --- | --- | --- |
| 1. Idealism3 | Pearson's r | — |  |  |  |  |  |  |
|  | p-value | — |  |  |  |  |  |  |
| 2. Idealism1 | Pearson's r | 0.395 | — |  |  |  |  |  |
|  | p-value | 7.132×10^-25^ | — |  |  |  |  |  |
| 3. Pos_Ultimate3 | Pearson's r | 0.39 | 0.371 | — |  |  |  |  |
|  | p-value | 3.207×10^-24^ | 5.463×10^-22^ | — |  |  |  |  |
| 4. Panpsychism3 | Pearson's r | 0.344 | 0.323 | 0.333 | — |  |  |  |
|  | p-value | 6.840×10^-19^ | 1.061×10^-16^ | 8.696×10^-18^ | — |  |  |  |
| 5. Idealism2 | Pearson's r | 0.391 | 0.212 | 0.191 | 0.272 | — |  |  |
|  | p-value | 1.999×10^-24^ | 7.981×10^-8^ | 1.485×10^-6^ | 3.613×10^-12^ | — |  |  |
| 6. Panpsychism2 | Pearson's r | 0.385 | 0.336 | 0.405 | 0.32 | 0.207 | — |  |
|  | p-value | 1.194×10^-23^ | 4.187×10^-18^ | 2.903×10^-26^ | 2.067×10^-16^ | 1.655×10^-7^ | — |  |
| 7. Dualism2 | Pearson's r | 0.406 | 0.412 | 0.347 | 0.37 | 0.242 | 0.431 | — |
|  | p-value | 2.674×10^-26^ | 3.741×10^-27^ | 3.131×10^-19^ | 8.646×10^-22^ | 7.247×10^-10^ | 7.416×10^-30^ | — |
| 8. Dualism3 | Pearson's r | 0.208 | 0.283 | 0.206 | 0.239 | 0.151 | 0.283 | 0.293 |
|  | p-value | 1.371×10^-7^ | 4.638×10^-13^ | 1.828×10^-7^ | 1.374×10^-9^ | 1.426×10^-4^ | 4.801×10^-13^ | 6.012×10^-14^ |

*Table A4. Bivariate correlations of the Materialism items*

| **Variable** |  | **Scientism1** | **Scientism3** | **Materialism1** | **Scientism2** | **Materialism2** | **Free_will2** | **Ultimate3** |
| --- | --- | --- | --- | --- | --- | --- | --- | --- |
| 1. Scientism1 | Pearson's r | — |  |  |  |  |  |  |
|  | p-value | — |  |  |  |  |  |  |
| 2. Scientism3 | Pearson's r | 0.63 | — |  |  |  |  |  |
|  | p-value | 6.102×10^-71^ | — |  |  |  |  |  |
| 3. Materialism1 | Pearson's r | 0.517 | 0.477 | — |  |  |  |  |
|  | p-value | 2.414×10^-44^ | 3.947×10^-37^ | — |  |  |  |  |
| 4. Scientism2 | Pearson's r | 0.594 | 0.55 | 0.543 | — |  |  |  |
|  | p-value | 3.578×10^-61^ | 5.832×10^-51^ | 1.428×10^-49^ | — |  |  |  |
| 5. Materialism2 | Pearson's r | 0.516 | 0.429 | 0.53 | 0.445 | — |  |  |
|  | p-value | 4.493×10^-44^ | 1.555×10^-29^ | 7.014×10^-47^ | 5.567×10^-32^ | — |  |  |
| 6. Free_will2 | Pearson's r | 0.443 | 0.455 | 0.546 | 0.43 | 0.507 | — |  |
|  | p-value | 1.129×10^-31^ | 1.747×10^-33^ | 3.811×10^-50^ | 9.812×10^-30^ | 1.801×10^-42^ | — |  |
| 7. Ultimate3 | Pearson's r | 0.375 | 0.425 | 0.5 | 0.457 | 0.348 | 0.418 | — |
|  | p-value | 1.965×10^-22^ | 6.367×10^-29^ | 4.393×10^-41^ | 7.399×10^-34^ | 2.719×10^-19^ | 4.853×10^-28^ | — |
| 8. Materialism3 | Pearson's r | 0.453 | 0.458 | 0.409 | 0.326 | 0.411 | 0.412 | 0.32 |
|  | p-value | 4.385×10^-33^ | 5.612×10^-34^ | 9.468×10^-27^ | 4.984×10^-17^ | 4.762×10^-27^ | 3.849×10^-27^ | 1.899×10^-16^ |

**References**

Epskamp, S., Borsboom, D., & Fried, E. I. (2018). Estimating psychological networks and their accuracy: A tutorial paper. *Behavior Research Methods*, *50*(1), 195–212. https://doi.org/10.3758/S13428-017-0862-1/FIGURES/9

Epskamp, S., Cramer, A. O. J., Waldorp, L. J., Schmittmann, V. D., & Borsboom, D. (2012). qgraph: Network Visualizations of Relationships in Psychometric Data. *Journal of Statistical Software*, *48*, 1–18. https://doi.org/10.18637/JSS.V048.I04

Foygel, R., & Drton, M. (2010). Extended Bayesian Information Criteria for Gaussian Graphical Models. *Advances in Neural Information Processing Systems 23: 24th Annual Conference on Neural Information Processing Systems 2010, NIPS 2010*. Retrieved from https://arxiv.org/abs/1011.6640v1

Friedman, J., Hastie, T., & Tibshirani, R. (2008). Sparse inverse covariance estimation with the graphical lasso. *Biostatistics (Oxford, England)*, *9*(3), 432–441. https://doi.org/10.1093/BIOSTATISTICS/KXM045

Fruchterman, T. M. J., & Reingold, E. M. (1991). Graph drawing by force-directed placement. *Software: Practice and Experience*, *21*(11), 1129–1164. https://doi.org/10.1002/SPE.4380211102

Haslbeck, J. M. B., & Waldorp, L. J. (2015). mgm: Estimating Time-Varying Mixed Graphical Models in High-Dimensional Data. *Journal of Statistical Software*, *93*, 1–46. https://doi.org/10.18637/JSS.V093.I08

Liu, H., Lafferty, J., & Wasserman, L. (2009). The Nonparanormal: Semiparametric Estimation of High Dimensional Undirected Graphs. *Journal of Machine Learning Research*, *10*, 2295–2328.

Zhao, T., Liu, H., Roeder, K., Lafferty, J., & Wasserman, L. (2012). The huge package for high-dimensional undirected graph estimation in R. *Journal of Machine Learning Research*, *13*, 1059–1062.
